# Lyprinol—Is It a Useful Anti-Inflammatory Agent?

**DOI:** 10.1093/ecam/nep030

**Published:** 2011-08-11

**Authors:** Sheila A. Doggrell

**Affiliations:** School of Life Sciences, Queensland University of Technology, GPO Box2434, Brisbane, QLD 4001, Australia

## Abstract

The New Zealand green lipped mussel preparation Lyprinol is available without a prescription from a supermarket, pharmacy or Web. The Food and Drug Administration have recently warned Lyprinol USA about their extravagant anti-inflammatory claims for Lyprinol appearing on the web. These claims are put to thorough review. Lyprinol does have anti-inflammatory mechanisms, and has anti-inflammatory effects in some animal models of inflammation. Lyprinol may have benefits in dogs with arthritis. There are design problems with the clinical trials of Lyprinol in humans as an anti-inflammatory agent in osteoarthritis and rheumatoid arthritis, making it difficult to give a definite answer to how effective Lyprinol is in these conditions, but any benefit is small. Lyprinol also has a small benefit in atopic allergy. As anti-inflammatory agents, there is little to choose between Lyprinol and fish oil. No adverse effects have been reported with Lyprinol. Thus, although it is difficult to conclude whether Lyprinol does much good, it can be concluded that Lyprinol probably does no major harm.

## 1. Introduction

The New Zealand green lipped mussel preparation Lyprinol is readily available without a prescription from a supermarket, pharmacy or web. Over the years, “Miracle from the sea”, and many other claims have been made about the therapeutic benefits of Lyprinol [1]. In 1999, there was the extraordinary situation of Lyprinol being promoted and marketed as an anti-cancer agent [2]. As a consequence, patients bought large quantities of this product at significant expense (NZ$2 million), despite there being no evidence of efficacy [3]. The Therapeutics Goods Administration in Australia advised cancer patients not to rely on this substance for the treatment of their cancer, and to seek medical advice.

More recently, Lyprinol has been promoted for its anti-inflammatory effects. The starting point for the interest in the green lipped mussel as therapy for inflammation was folklore. This folklore was that coastal dwelling Maori in New Zealand, who regularly consumed mussels as part of their diet, suffered far less from the ravages of arthritis than their inland dwelling relatives [1].

The trade mark for Lyprinol is held by Pharmalink, who state that “Lyprinol has been usefully for the treatment of osteoarthritis, rheumatoid arthritis, asthma and gout and is being studied for its effectiveness against the other inflammatory diseases such as Crohn's disease, ulcerative colitis, lupus, psoriasis and others” [4]. Lyprinol is marketed on the net by NZ Nutraceuticals, which states it has “clinically proven anti-inflammatory properties”, and that “as an effective anti-inflammatory agent, Lyprinol is used to treat arthritis, osteoarthritis, rheumatoid arthritis, asthma, muscle pain (particularly for sports people)” [5]. Lyprinol is marketed in Australia by Blackmores, and the packet states “Its potent anti-inflammatory actions assist in the maintenance of healthy airways and breathing passages, as well as providing relief from joint swelling and arthritis.”

As recently as 2007, The Food and Drug Administration warned LyprinolUSA about their extravagant anti-inflammatory claims for Lyprinol appearing on the web [6]. LyprinexTM also contains Lyprinol and is marketed internationally, including on the web by Life Plus International [7]. LyprinexTM claimed “Lyprinol has been shown to improve the following conditions: Inflammation; Pain due to inflammation; Bronchial tightness; Allergy symptoms. Additional benefits: (Lyprinol) May lower depression: May reduce the risk of coronary heart disease. It (Lyprinol) improves the condition of patients with rheumatoid arthritis: it improves the condition of patients with asthma [7].”

There were some initial problems with extracting the ingredients from *Perna canaliculus*, the NZ green lipped mussel, as most of the activity was lost with heat treatments or freeze drying [1]. Thus, some of the early studies may have been carried out with preparations with no active ingredients, and (not surprisingly), an anti-inflammatory action was not consistently reported [1]. The process now being used to extract the ingredients from the mussels does result in Lyprinol having active ingredients [8]. As these extraction problems were sorted out by 1990, only publications from 1990 onwards are considered in this review. In addition to Lyprinol (also known as Seatone) and products derived from Lyprinol, Perna (the lyophilized *P. canaliculus* powder) is active, and is discussed in this review.

Mobicosa is a new freeze-dried preparation of the green lipped mussel which in addition to the fatty acids reported in Lyprinol (see Section 2) contains other natural agents (glucosamines and chrondroitin sulphates) [9]. These compounds have been claimed to have benefits in arthritis in their own right. Consequently, it is difficult to determine whether any benefits of Mobicosa are due to the fatty acids in the *P. canaliculus*, or the glucosamines and chrondroitin. For this reason, and also because, to my knowledge, there are no published studies of the effects of Mobicosa in clinical trials in animals or humans, Mobicosa is not included in this review.

This review is a thorough review of Lyprinol as an anti-inflammatory agent. Pubmed and the Internet were searched for references to Lyprinol, Seatone and Perna alone, available from 1990 onwards. In the first part of the review, the anti-inflammatory mechanisms of actions of Lyprinol/Seatone/Perna are discussed. This is followed by a review of the evidence for anti-inflammatory effects with Lyprinol/Seatone/Perna in animal models. The major emphasis of this review is the clinical trials with Lyprinol, which are critically discussed. Fish oil is another complementary medicine that is used as an anti-inflammatory agent. Fish oil has some of the same ingredients as Lyprinol, and the final part of the review considers studies that have compared fish oil and Lyprinol as anti-inflammatory agents. The search words for this final part of the review are fish oil and Lyprinol/Seatone/Perna and inflammation or anti-inflammatory.

## 2. Contents of Lyprinol

Lyprinol is a mixture of the five main lipid classes including sterol esters, triglycerides, free fatty acids, sterols and polar lipids [1]. Lyprinol contains two of the long chain omega-3 polyunsaturated fatty acids (PUFAs); eicosapentaenoic acid (EPA) and docosahexaenoic acid (DHA) [1]. In amounts, Lyprinol is 13% EPA, 21% DHA and about 30% cholesterol [10]. Additionally, Lyprinol contains some novel *ω*-3 PUFAs; 5,9,12,15-octodecatretraenoic acid, 5,9,12,16-nondecatertraenoic acid, 7,11,14,17-eicosatetraenoic acid, and 5,9,12,15,18-heneicsapententaenoic acid [11]. EPA and DHA are the main ingredients of fish oil supplements. Thus, there will be similarities between Lyprinol and fish oil, which are discussed later in this review.

EPA, DHA and the *ω*-3 PUFA 7,11,14,17-eicosatetraenoic acid are similar in structure to arachidonic acid (5,8,11,14-eicosatraenoic acid), the precursor to the inflammatory agents, prostaglandins and leukotrienes. Thus, it is probably not surprising that Lyprinol can modulate the effects of these inflammatory agents to exert an anti-inflammatory effect.

## 3. Anti-Inflammatory Mechanisms of Action of Lyprinol

As competitive substrates for the cyclooxygenase enzyme (COX; synthesis of prostaglandins) and the lipoxygenase enzyme (synthesis of leukotrienes), EPA and DHA reduce the levels of the inflammatory prostaglandins and leukotrienes. Lyprinol also does this, presumably (at least partly) due to the EPA and DHA content. In human monocytes, PGE_2_ production from arachidonic acid was inhibited by Lyprinol with an IC_50_ of 1.2 *μ*g mL^−1^ [12]. With human polymorphonuclear leukocytes in the presence of arachidonic acid, Lyprinol (100 *μ*g mL^−1^) abolished the formation of leukotrienes (LT) B_4_ and reduced the formation of 5-HETE (products from the lipooxygenase pathways) [12]. The free fatty acids from Lyprinol have also been shown to inhibit the formation of LTB_4_ and 5-HETE from human neutrophils stimulated with arachidonic acid and a calcium ionophore [11].

Lyprinol also has a direct ability to inhibit the COX enzymes (COX-1 and COX-2) [13]. COX-2 is the inducible enzyme commonly associated with excessive inflammation. Thus, *P. canaliculus* at 1 *μ*g mL^−1^ inhibited COX-1 and COX-2 by 12% and 25%, respectively [13]. After hydrolysis to the free fatty acid fraction, inhibition was increased to 49% for COX-1 and 60% for COX-2, and when the free fatty acids were separated, inhibition was increased further to 78% for COX-1 and 70% for COX-2 [13]. Both the Tween-20 extract and the glycogen extract of Perna (Aroma NZ Ltd), which is lyophilized *P. canaliculus* powder, have been shown to inhibit COX-1 and COX-2 [14].

In addition to prostaglandins and leukotrienes, histamine and cytokines are mediators of inflammation. In 1986, there was a brief report of anti-histaminic activity with *P. canaliculus* [15]. Recently, Lyprinol has been shown to decrease the ability of lipopolysaccharide (LPS) to stimulate tumour necrosis factor-alpha (TNF-*α*) and interferon-gamma (IFN-*γ*) in splenocytes from a rat model of arthritis, where Freund's complete adjuvant containing *Mycobactrium butyricum* was injected into the paw [16]. The levels of TNF-*α* and IFN-*γ* were raised in untreated arthritis to 3.1 and 10.7 ng mL^−1^, and after Lyprinol treatment were decreased to 1.71 and 3.0 mg mL^−1^, respectively [16].

Perna has also been shown to reduce the concentrations of TNF-*α* and interleukin (IL)-12p40 production from LPS stimulated human THP-1 monocytes [17]. In addition to inhibiting TNF-*α*, Tween-20 extracts of Perna have been shown to inhibit the production of IL-1, IL-2 and IL-6 in isolated cell preparations, and also to inhibit IgG production [14].

Recently, proteomics in the splenocytes from the rat model of arthritis induced by *M. butyricum* has shown changes in protein expression with Lyprinol, but it is not clear whether these changes relate to the anti-inflammatory effect of Lyprinol. Thus, Lyprinol changed the expression of several proteins related to metabolism (increased expression of malate dehydrogenase, and decreased expression of protein-*o*-mannosyl-transferase 2,titin-cap protein, and protein disulfide isomerase) and decreased expression of Tdrd7, telethonin and dynactin [18]. The authors hypothesize that these changes in proteins related to metabolism may be responsible for the anti-inflammatory effects of Lyprinol. Many further experiments are required to test this hypothesis including testing whether Lyprinol alters the expression of these proteins in other models of inflammation, and what, if any, is the relationship between these proteins and the anti-inflammatory effects of Lyprinol.

## 4. Lyprinol in Animal Models of Arthritis

### 4.1. Carrageenan Model

In one of the standard models of arthritis used in experimental studies of arthritis, the injection of carrageenan into rear paws of rats to induce swelling, Lyprinol showed little activity [12]. However, Lyprinol has been shown to reduce swelling in other animal models of arthritis.

### 4.2. Collagen Type-II-Induced Arthritis

Collagen type-II-induced arthritis is another established animal model of human arthritis, and in this model, Lyprinol does suppress inflammation [12]. In this model, the collagen is emulsified with complete Freund's adjuvant and injected into the rear right paw of the rat [12]. The amounts of paw swelling were 1.77 and 1.62 mm in the rear left and right rear paws, respectively, and this was reduced to 0.32 and 1.04 mm with Lyprinol (20 mg kg^−1^) and to 0.82 and 1.48 mm with ibuprofen (50 mg kg^−1^) [12].

Glucocorticoids are the standard treatment of severe arthritis, and when new drugs are developed for arthritis, they are compared to the glucocorticoids, as only if agents can be shown to be better or additive with glucocorticoids are they likely to have a role in clinical practice. When collagen type-II in Freund's incomplete adjuvant was used to induce arthritis in rats, Lyprinol at 20 mg kg^−1^ had no effect alone on paw swelling, nor did the glucocorticoid prednisone at 2.5 mg kg^−1^ alone [19]. However, when these agents were combined, at the same doses, the paw swelling was reduced [19]. Key studies comparing Lyprinol to an effective dose of glucocorticoid, or with an effective dose of glucocorticoid, have not been reported in this standard model or arthritis.

Perna has also been tested in the collagen type-II-induced severe polyarticular arthritis model in the rat and mouse [17]. In the rat, when Perna 100 mg kg^−1^ day^−1^ treatment was started at the same time as the collagen type II, there was a reduction in the incidence of developing arthritis from 58% in the control group to 17% in the Perna group [17]. In the mouse with established arthritis, Perna had no effect on the arthritis scores after 10 days, but reduced the score by day 81 [17].

### 4.3. Zymosan-Induced Inflammation

Zymosan is commonly used as a local irritant to cause acute inflammation. Pentoxifylline acts to reduce the levels of the inflammatory interleukins and TNF-*α*. Lyprinol had an additive effect with pentoxifylline in rat zymosan models of arthritis. Thus, in zymosan-induced paw swelling, pentoxifylline (125 mg kg^−1^) and Lyprinol (20 mg kg^−1^) alone reduced paw swelling by 15–20%, and in combination by about 50% [20].

### 4.4. *Mycobacterium tuberculosis*

When chronic polyarthritis was induced in rats using adjuvant, prepared from dried *M. tuberculosis*, inoculated into the tailbase, Lyprinol pre-treatment (20 mg kg^−1^ with continued treatment) reduced paw swelling [12]. The level of swelling in the front and rear paw group after adjuvant treatment was 2.8 mm and 1.2 mm, and this was reduced to 1.4 mm and 0.23 mm, respectively, by Lyprinol [12]. Lyprinol 20 mg kg^−1^ had a greater effect in reducing paw swelling in this model of arthritis than the standard non-steroidal anti-inflammatory agents: aspirin (300 mg kg^−1^), ibuprofen (40 mg kg^−1^) or naproxen (25 mg kg^−1^) [12].

When arthritis was induced in rats using adjuvants prepared from dried *M. tuberculosis*, Lyprinol alone at 20 mg kg^−1^ had little effect, and the glucocorticoid prednisone at 2.5 or 10 mg kg^−1^ had no effect alone [19]. This suggests that this is a relatively glucocorticoid-insensitive model of arthritis. However, when the lower dose of prednisone was combined with Lyprinol there was a reduction in paw swelling, and this reduction was greater than with Lyprinol alone [19]. The COX inhibitors, aspirin (200 mg kg^−1^), diflunisal (80 mg kg^−1^) and mefenamic acid (150 mg kg^−1^) were also ineffective alone in this model, but were effective when combined with Lyprinol 20 mg kg^−1^ [19]. The free fatty acids of *P. canaliculus* mussel powder at 30 mg kg^−1^ have recently been to have a similar ability to piroxicam (2 mg kg^−1^) to reduce the swelling induced by adjuvants prepared from dried *M. tuberculosis* [21].

Pain was the main focus of a study where arthritis was induced in rats by injecting Freund's complete adjuvant containing *M. butyicum* into the hind paw, Lyprinol 25 mg kg^−1^ reduced the swelling [16]. Associated with this reduction in swelling, there was initially a similar reduction in pain score (vocalizations during flexions of the paw) with Lyprinol and naproxen (20 mg kg^−1^) [16]. The pain reduction was maintained in the presence of naproxen but not Lyprinol [16]. In splenocytes from this model at 14 days, Lyprinol and naproxen treatment were associated with a similar reduced ability of LPS to stimulate TNF-*α* and IFN-*γ* [16].

Pentoxifylline (125 mg kg^−1^) and Lyprinol (20 mg kg^−1^) alone had no significant effect on the swelling in pre-established *M. tuberculosis* induced arthritis to a small extent, induced arthritis, whereas in combination the swelling was reduced to a small extent [20].

## 5. Lyprinol in Other Animal Models of Disease

There is some preliminary evidence that Lyprinol may be potentially useful in ameliorating the symptoms of inflammatory bowel disease. Thus, in a mouse model of inflammatory bowel disease induced by dextran sulphate sodium, Lyprinol (Pharmalink International) 5 mg per day reduced body weight loss, decreased disease activity in the colon, and reduced the distal colon crypt area losses [22].

Intestinal mucositis is a common and debilitating side effect of some kinds of chemotherapy including that with 5-fluorouracil. In a rat model where 5-fluorouracil was used to cause intestinal mucositis, Lyprinol prevented weight loss and histological damage severity [23].

Lyprinol has also been shown to reduce the contractions of the isolated rat uterus, and it has been suggested that it may be useful for the treatment of dysmenorrhoea [24]. An advantage Lyprinol had over the NSAIDs (aspirin, ibuprofen and naproxen), was that it did not induce gastric lesions in rats [24].

## 6. Studies of Lyprinol in Dogs with Arthritis

Studies have shown that Lyprinol is effective in treating dogs with arthritis. In 2001, it was shown that when dogs with osteoarthritis were fed with *P. canaliculus*, there was an improvement compared to untreated dogs [25]. Three placebo-controlled studies involving 96 arthritic dogs were undertaken where the Perna (450, 750 and 1000 mg for dogs <25, 25–34 and >34 kg, respectively) was added to the top of standard food, incorporated into a treat or incorporated into the food [25]. All the studies had similar findings; showing that after 6 weeks, compared to placebo, Lyprinol was beneficial. Arthritis scores were of mobility, and the pain, swelling and crepitus of individual joints and of each limb. When *P. canaliculus* was added to the standard food, it reduced the scores compared to the untreated dogs [25]. When the individual items of the arthritis scores were separated, it was shown that there was a major decrease in the pain score and a modest reduction in joint swelling and crepitus [25].

However, these results were not substantiated in a 12-week trial comparing Green shell mussel powder (11 mg) to chondroitin sulphate and placebo in 58 dogs with degenerative joint disease of the shoulder, elbow, hip joints, and/or stifle [26]. Both owners and veterinarians reported a slight improvement of the symptoms in all three groups (i.e. including the placebo group) [26]. It has been suggested that the mussel extract was ineffective in this study due to the low dose [27].

A further study showed that at 125 mg, Green lipped muscle extract was effective in dogs with degenerative joint disease [27]. This trial used 81 lame dogs, compared the mussel extract to placebo, and assessed the severity of musculoskeletal dysfunction [27]. Although there was no improvement after 28 days, by 56 days, 67% of the lame dogs were showing improvement in the mussel group, compared to 41% in the placebo group [27]. However, by day 112, there was no significant difference between the groups [27]. No adverse effects or toxicity was noted with the mussel extract in dogs [27].

In 2007, Perna was compared to placebo and the non-steroidal anti-inflammatory drug carprofen in 45 dogs with chronic pain due to osteoarthritis [28]. In this study, the placebo group showed a 20–40% improvement in pain/chronic pain, the veterinary mobility index, locomotion, and force exerted by the most effected leg, after 8 weeks [28]. The pain VAS improved by 67% with Perna and 86% with the non-steroidal anti-inflammatory drug carprofen [28]. Carprofen was also more effective than Perna in improving the force exerted by the leg (67% versus 47%; placebo 27%) [28]. Perna and carprofen caused similar improvements in chronic pain (80%; placebo, 20%) and the veterinary mobility index (67%; placebo, 27%) [28].

## 7. Clinical Trials with Lyprinol in Humans

Clinical trials of the effects of Lyprinol in subjects with arthritis and asthma have been reported, and are described in this section. The question, this part of the review asks, is whether Lyprinol has been shown to be anti-inflammatory in humans? After searching and collecting all the information found on Pubmed and the Internet for Lyprinol/Seatone/Perna from 1990 onwards, the clinical trials in inflammation were selected, and all of these are reviewed. Most of the clinical trials with Lyprinol have flaws. It was decided not to exclude any of these, but to present the results of each clinical trial in sequence, with a discussion of any problems with the methodology and interpretation of results in the trial.

To my knowledge, there are no ongoing clinical trials with Lyprinol. Some clinical trials that have previously been reported to be in progress (Crohn's disease, ulcerative colitis, lupus, psoriasis), but have not been reported. The clinical trials presented show that there is some evidence that Lyprinol may have a small efficacious effect in osteoarthritis and rheumatoid arthritis.

### 7.1. Osteoarthritis and Rheumatoid Arthritis

In 1998, the effects of the stabilized green-lipped mussel powder 1150 mg per day (five capsules, Group A) was compared to that of the lipid extract 210 mg per day (three capsules, Group B) in subjects with osteoarthritis and rheumatoid arthritis at the outpatients' clinic of the Glasgow Homoeopathic Hospital [29]. Both groups (A and B) had 15 subjects with osteoarthritis and 15 subjects with rheumatoid arthritis [29]. Most of the subjects with osteoarthritis were taking non-steroidal anti-inflammatory drugs, while half of the subjects with rheumatoid arthritis were taking second line drugs [29], which were not specified. In osteoarthritis, there were improvements after 3 months of articular index, morning stiffness and functional index with both preparations. Thus, in subjects with osteoarthritis in Group A, the articular index decreased from a baseline of 9.5 to 4.3, morning stiffness from 52.5 to 24.3, and the functional index from 10.4 to 4.8 [29]. In subjects with rheumatoid arthritis in Group A, articular index decreased from a baseline of 14.8 to 5.9, morning stiffness from 98.4 to 27.1, and the functional index from 12.9 to 6.9 [29]. Similar results for these parameters were obtained in Group B. Similar reductions in pain scores were obtained in the subjects with osteoarthritis in Group A (6.1 to 4.8) and Group B (6.1 to 5.0), but this was only significant in Group B [29]. In rheumatoid arthritis, both preparations of Lyprinol improved articular index, morning stiffness and functional index to a similar extent as in osteoarthritis, and there was no significant improvement in pain scores [29]. Neither preparation improved grip strength in the right or left hands of subjects with either osteoarthritis or rheumatoid arthritis [29].

A major flaw in this trial was that there was not a placebo group, and we will never know for certain whether these effects of the Lyprinol were greater than the placebo effect. The authors argue that the placebo effect is small in arthritis [29], but without a placebo group we cannot tell whether this would have been so in this protocol. Recent placebo-controlled trials suggest that there is about a 20% reduction in osteoarthritis symptoms with placebo. Thus, in a 12-week placebo-controlled trial of tramadol in osteoarthritis of the knee, there was about a 20% reduction in pain, stiffness and physical function with placebo [30]. In trials of diacerein and othokin in osteoarthritis, the placebo also reduced pain by about 20% at 3 months [31, 32]. The reductions in articular index, morning stiffness and functional index with the mussel powder or lipid extract were about 55%, which (if we accept 20% as the placebo level) suggests that Lyprinol does have benefits on these symptoms. Lyprinol reduced pain by 20% in the clinical trial with the mussel powder or lipid extract [29], which is comparable to the placebo effect in placebo-controlled trials in osteoarthritis. This suggests that Lyprinol is having little or no effect on the pain associated with osteoarthritis.

### 7.2. Osteoarthritis

There was another study suggesting that Lyprinol was effective in relieving the pain osteoarthritis, published in 2003. All 54 patients heard detailed information about Lyprinol, and were treated with Lyprinol (two capsules bid) [33]. After 8 weeks, of 56 subjects with hip and knee osteoporosis, there was a reduction in pain from 6.4 cm on the Visual Analogue Scale to 3.9 [33], a 39% reduction. A similar percentage benefit was observed with Lyprinol in joint function [33]. At 8 weeks, 87% of patients and 90% of doctors reported improved in global assessment [33].

To be properly controlled, this clinical trial should have provided information to all participants about placebo, and compared Lyprinol with a control group receiving a placebo. Without this control group it is difficult to determine whether Lyprinol was effective, or it was a combination of the information and placebo effect that was effective in the group treated with Lyprinol. There is also a discrepancy between this study showing a reduction in pain of 39% with Lyprinol in osteoarthritis [33] to the previous study showing a reduction in pain of 20% (discussed in previous section [29]), which is equivalent to the placebo response. There are several possible reasons for this discrepancy. For instance, the doses of Lyprinol are not in the same range but not identical in the two studies, and the length of the studies are different (3 months versus 8 weeks). Another reason for the discrepancy could be that there was a positive response to the information in the Lyprinol group.

The final trial of Lyprinol in osteoarthritis was placebo-controlled and reported in 2004 [33]. In this trial, 80 subjects with knee osteoarthritis were randomized on a double-blind manner to Lyprinol or placebo [34]. Pain scores decreased in both groups, and were lower for Lyprinol than placebo after 8 and 12 weeks (VAS of *∼*51 mm versus 57 mm at both time points), but not 18 weeks (VAS of *∼*53 versus 56) [34]. The patients' global assessment of their arthritis showed improvement in both groups, and was only significant greater for Lyprinol after 12 (2.8 versus 3.2 in the placebo group) and 18 weeks (3.0 versus 3.1) [34]. However, there was no difference in the physicians' global assessment of arthritis between groups [34]. In this study, the reductions in pain scores and the patients' assessment of improvement with Lyprinol were quite small. Also, as both groups were allowed to take unlimited paracetamol, there was great variability in the intake of paracetamol in both groups calculated as a percentage of baseline intake [34]. Without the absolute amounts of paracetamol taken by the subjects, it difficult to assess whether paracetamol contributed to the small reduction in pain observed with Lyprinol.

### 7.3. Rheumatoid Arthritis

Lyprinol in combination with fish oil has been tested in 50 adults with rheumatoid joint disorder, moderate pain and morning stiffness [35]. After 12 weeks of treatment, the mean duration of morning stiffness was reduced from 13.7 to 12.4 min, the number of painful joints from 4.18 to 3.58, and the number of swollen joints from 2.62 to 1.94 by the combination of Lyprinol and fish oil [35]. Pain was evaluated separately by the patients and physicians, and was also reduced with the combination treatment [35].

The two most obvious criticisms of this study, is that there was no placebo group, and thus we do not know whether the small benefits observed were a placebo response or due to the combination of Lyprinol and fish oil. Secondly, if we concede that there may be a small beneficial response, we cannot determine whether it is due to Lyprinol, fish oil or the combination? For instance, if fish oil was effective, and Lyprinol was not, the combination could still show benefit.

### 7.4. Asthma

A small clinical trial suggests that Lyprinol may be useful against some of the symptoms of atopic (allergic) asthma. The 46 subjects in this trial has mild asthma with symptoms twice a week or less with a FEV_1_ (the forced expiratory volume in one second) of ≥80% of predicted, and were using short-acting *β*-adrenoceptor agonists for symptom relief [36]. Subjects receive either two tablets bid of either Lyprinol or placebo for 8 weeks. Lyprinol reduced daytime wheeze and improved morning peak expiratory flow, but did not improve night awakenings, use of *β*-agonists, or FEV_1_ [36]. It was suggested that the FEV_1_ was quite high in these subjects, and difficult to improve [36].

Glucocorticoids improve FEV_1_ in subjects with asthma. Thus, it seems unlikely that Lyprinol will replace the steroids in the treatment of mild asthma. A clinical trial determining the effect of Lyprinol on the FEV_1_ in subjects with more severe asthma would be on interest. One point in favour of the use of Lyprinol is that the small benefits come with no excess of adverse effects [36].

In their labelling, Blackmores have emphasized that there is good clinical evidence that Lyprinol causes a small improvement in lung function, rather than any beneficial effects in arthritis. This labelling is supported by the clinical trials with Lyprinol reported here.

## 8. Studies Comparing the Anti-Inflammatory Effects of Lyprinol and Fish Oil

EPA and DHA are the main constituents of fish oil supplements. Fish oil is probably anti-inflammatory because, like Lyprinol, it inhibits the production of eicosanoids and cytokines [37]. Recently, a direct comparison of Lyprinol and fish oil preparations (Blackmore's), available in Australia, has shown EPA (1 *μ*g mL^−1^) inhibits COX-1 and COX-2 by 92% and 91%, and DHA inhibits by 65% and 95%, respectively [13]. These values are mainly higher than the inhibition observed with Lyprinol and its free fatty acid extracts (values given previously), probably because the levels of EPA and DHA in Lyprinol are lower than in preparations of EPA or DHA [13]. However, when Lyprinol is hydrolysed it has a similar ability to inhibit the COX enzymes as fish oil [13].

The evidence that fish oil is anti-inflammatory when used clinically as an adjunct in rheumatoid arthritis is quite good [38]. However, the evidence for clinical efficacy for fish oil in osteoarthritis and asthma is weak [37].

There is a clinical trial showing that the combination of Lyprinol, EPA, and DHA are effective and well-tolerated in the treatment of rheumatoid arthritis [34]. Unfortunately, the effects of the combination were not compared to Lyprinol alone, or EPA/DHA alone, in this study, making it impossible to determine whether any of the benefit was due to Lyprinol, or whether Lyprinol has added benefits to EPA/DHA. Thus, head to head trials of Lyprinol and fish oil should be undertaken in subjects with rheumatoid arthritis, preferable with a placebo group.

In a mouse model of inflammatory bowel disease, Lyprinol 5 mg/day prevented body weight loss whereas EPA/DHA 55 mg/day did not [22]. Lyprinol was also more effective than the EPA/DHA in reducing disease activity index, which included body weight loss, rectal bleeding, stool consistency, and overall condition [22]. Lyprinol increased the crypt area index, whereas EPA/DHA did not [22]. To date, the clinical trials of Lyprinol in inflammatory bowel disease have not been reported. Clinical trials of fish oil in Crohn's disease [39] and ulcerative colitis [40] have not shown clear cut benefits. A clinical trial of Lyprinol alone in inflammatory bowel disease is indicated.

In summary, there is weak evidence that fish oil and Lyprinol have a small benefit in osteoarthritis, rheumatoid arthritis, and asthma, and as clinical anti-inflammatory agents there is little to choose between them. Fish oil does have the benefit of being antithrombotic, and reducing the incidence of coronary heart disease and stroke. Thus, in subjects with arthritis at high cardiovascular risk, fish oil may have a better overall effect than Lyprinol.

## 9. Conclusion

The experimental evidence that Lyprinol has anti-inflammatory effects is good, and consequently it may work in humans. There is lot of hype about the clinical uses of Lyprinol on the Web (and in the wider media over the years), but much of it is not supported by good clinical trials. Appropriate clinical trials have not been undertaken. For instance, both randomized, double-blind, placebo-controlled trials and comparator trials with standard drugs need to be undertaken, with Lyprinol in rheumatoid arthritis and osteoarthritis. Without good clinical trials, we cannot conclude that Lyprinol is effective or ineffective in human inflammation. No adverse effects have been reported with Lyprinol. Thus, although it is difficult to conclude from the completed clinical trials whether it does much good, it can be concluded that Lyprinol probably does no harm.
